# Bionic Design and Optimization on the Flow Channel of a Legged Robot Joint Hydraulic Drive Unit Based on Additive Manufacturing

**DOI:** 10.3390/biomimetics9010013

**Published:** 2023-12-31

**Authors:** Zhipeng Huang, Chenhao Du, Chenxu Wang, Qianran Sun, Yuepeng Xu, Lufang Shao, Bin Yu, Guoliang Ma, Xiangdong Kong

**Affiliations:** 1 School of Mechanical Engineering, Yanshan University, Qinhuangdao 066004, China; zhipengh@stumail.ysu.edu.cn (Z.H.); dch@stumail.ysu.edu.cn (C.D.); wcx1@stumail.ysu.edu.cn (C.W.); sqr@stumail.ysu.edu.cn (Q.S.); chunhao@stumail.ysu.edu.cn (Y.X.); magl@ysu.edu.cn (G.M.); xdkong@ysu.edu.cn (X.K.); 2 School of Art and Design, Yanshan University, Qinhuangdao 066004, China; slf2021666@stumail.ysu.edu.cn; 3 Hebei Provincial Key Laboratory of Heavy Machinery Fluid Power Transmission and Control, Qinhuangdao 066004, China

**Keywords:** joint hydraulic drive unit, incremental technique, pressure loss, bionic flow channel

## Abstract

The joint hydraulic drive unit (HDU) serves as a pivotal element in enabling the high-performance movements of legged robots. Functioning as the conduit linking the oil source and the actuator, the hydraulic flow channel significantly impacts actuator performance. Hence, optimizing the HDU flow channel becomes imperative, enhancing not only HDU efficiency but also the overall system performance. This paper introduces a novel approach by aligning the hydraulic flow channel of the joint HDU with the arteriovenous layout of the cardiac vascular system, departing from the conventional machining flow channel model. Through simulations determining the optimal range of the vascular branch radius and angle, this study guides the design optimization of the joint HDU flow channel. With the primary optimization goal of reducing pressure loss, the study compares simulation outcomes of various flow channel models—linear, variable excessive radius, and the multidimensional Bessel curve—tailored to suit the arrangement specifics of the joint HDU. Further validating these designs, the flow channels are fabricated using additive manufacturing for experimental verification. The integration of simulation analyses and pressure loss testing reveals a remarkable reduction of over 40% in pressure loss for the bionic flow channel compared to the conventional machining form. This empirical evidence strongly substantiates the bionic flow channel’s superior efficacy in pressure loss reduction. The findings presented herein offer valuable insights for the development of low-loss flow channels in joint HDUs, thereby presenting a new avenue for designing energy-efficient, high power-to-weight ratio legged robots.

## 1. Introduction

The robot joint HDU is mainly integrated by a hydraulic cylinder shell, a servo valve, a piston, a piston rod, an end cover, a sensor, a hydraulic pipeline, and other structures. Among them, the hydraulic pipeline transports oil for the actuator. Today, most of the hydraulic power units are also connected by hydraulic hoses. In the process of use, the intricate pipeline causes the hydraulic system to be messy and heavy, and it is also accompanied by problems such as a large linear loss and leakage [1,2]. Therefore, optimizing the hydraulic pipeline to reduce its pressure loss has become a problem studied by many scholars [3,4,5,6].

Compared with the traditional processing method, the forming method of additive manufacturing technology is top-down, and the geometry of the model is less demanding. For the flow channel, additive manufacturing can not only optimize the cross-section of the flow channel, but also optimize the connection mode of the flow channel. Therefore, additive manufacturing technology has been increasingly more applied in the field of hydraulic system manufacturing [7], and has brought new ideas to flow channel optimization [8]. For example, Zhang et al. [9] conducted a mathematical analysis of the flow channel of the valve block. By balancing the pressure loss and space requirements of the full-factor experimental method and combining the production advantages of additive manufacturing, a new way of constructing the pipeline could effectively reduce the pressure loss. Alshare et al. [10] carried out computational fluid dynamics analysis as well as fluid–solid coupling simulation based on the pressure distribution imported from CFD to determine the distribution of pipelines in the valve block. Finally, the weight of the valve block produced by additive manufacturing technology was reduced by 84%. The Lotus racing car and Red Bull Technology and Warwick Manufacturing Group [11] used additive manufacturing technology and Ti64 metal powder to process hydraulic manifold blocks and used a smooth flow channel at the right angle corner of the flow channel to reduce the energy consumption of the hydraulic system. Based on SLM technology, Timothy Simpson et al. [12] formed a hydraulic component with a complex inner cavity structure. The final part was not only lightweight, but the flow performance of the flow channel was also improved.

Many creatures in nature are constantly adaptively evolving in order to adapt to environmental changes [13,14]. With the in-depth study of bionics, human beings have found a variety of biological structures [15,16] in nature that are efficient and conform to the laws of thermodynamics and hydrodynamics. These structures provide a lot of inspiration for human design. Therefore, many people have used bionics to optimize the flow channel to reduce pressure loss, and bionics has been increasingly applied in fluid power technology [17]. Designing flow channels from a biomimetic perspective to reduce pressure losses and enhance heat dissipation is increasingly prevalent [18]. For example, biomimetic structures inspired by plant leaves [19] and V-shaped microstructures have been designed based on the human vascular system [20].

Because plants have to spend less energy to transport water to survive, natural branching systems such as crowns, plant roots [21,22], and veins have acted as bionic inspirations to people. For example, Roshandel et al. [23] combined the leaf vein layout with the straight channel to design a new flow channel structure, and the results of the numerical simulation showed that the use of this flow channel could significantly reduce energy loss. Xia et al. [24] designed a composite bionic flow channel based on the structure of veins and bird wings, which improved the performance of the fuel cell and prolonged its service life. Liang et al. [25] proposed a new permeability calculation model for a leaf vein fractal network, and discussed the influence of the channel aspect ratio, the Y-shaped bifurcation angle, and the total branch level number and damage number on effective permeability. Liu et al. [26] designed a liquid cooling plate with bionic vein branch channels, and the optimized channel could effectively alleviate the temperature rise of the battery. Zhu et al. [27] proposed a new bionic vapor chamber based on the porous composite structure of veins, which improved the heat transfer performance of the vapor chamber. Inspired by veins, Hu et al. [28] constructed a new type of bionic multicellular tube with stiffeners, which could significantly improve the ability of the shell to absorb shocks. Inspired by the lotus, Gong et al. [29] designed a new bionic multicellular tube with good impact resistance. Krishna et al. [30] studied a new type of bionic venation fin for metal hydride reactors, and proposed two reactor designs for heat transfer fluid flow. These designs provide better heat transfer and temperature uniformity. Lorenzini-Gutierrez et al. [31] proposed that increasing the number of stages in the flow channel is beneficial to improve the uniformity of fluid distribution and reduce the pressure loss in the flow channel. Chai et al. [32] drew inspiration from plant vein networks, employing their intricate hierarchical structure as a guiding principle to design liquid channels in VC. They examined the heat transfer and flow performance of both symmetric and asymmetric leaf vein (SLV and ALV) networks.

After long-term evolution, animal blood vessels have become a very mature nutrient delivery system. Moreover, the fluid transmission system is highly similar to the biological circulatory system in terms of composition, so many people design bionic flow channels for blood vessels [33]. For example, Li et al. [34] designed a three-dimensional bionic blood vessel network based on Murray’s law and used 3D printing to achieve its formation, which preliminarily verified that the 3D bionic design blood vessel showed significantly enhanced self-healing performance after injury. Misra et al. [35] determined that the shear stress of the arterial wall decreased with the increase in the bifurcation artery radius ratio in the process of arterial flow, and when the bifurcation angle increased, the wall shear stress became larger, which provided theoretical support for the study of the bifurcation angle of the bionic flow channel. Chen et al. [36] established a dynamic model of bionic pipelines by simulating the structure and mechanical properties of blood vessels and built a bionic pipeline test platform. The superiority of the bionic pipeline was verified by orthogonal testing. Based on the unique physiological structure of a cheetah’s heart, Quan L et al. [37] innovatively designed a bionic hydraulic pipeline with a three-layer structure, which had a good absorption effect on flow pulsation. Dang et al. [38] studied the process of water flow in a porous layer based on Murray’s law and a symmetrical bionic flow field, by analyzing the transport characteristics of water and provided suggestions for this type of flow field design.

From the studies mentioned above, it is evident that while some research has explored constructing channels from a biomimetic perspective, most have solely focused on plant roots and stems, overlooking the biomimetic study of the excellent transportation channels in the human vascular system. From this point of view, many studies have only considered the use of additive manufacturing technology and bionic design to achieve lightweight hydraulic components, and most of them have been aimed at valve block optimization, with a lack of the combination of the two to optimize the design of the HDU flow channel.

This paper analyzes the flow channel of the HDU. Firstly, the pipeline of the HDU is connected with the arteriovenous vascular layout of the cardiac vascular system. Then, in terms of the fractal and arrangement of the flow channel, based on the guidance of Murray’s law, the range of the branch angle and the radius ratio of the pipeline are determined. Then, the flow field mode and loss along the path at different angles are analyzed by ANSYS 2020, and the flow channel is improved. A flow channel layout method suitable for the characteristics of additive manufacturing technology is proposed, and finally the effectiveness of bionic flow channels in reducing pressure loss by building a pressure loss test platform is presented.

## 2. Establishment of Key Parameter Model of the Flow Channel

### 2.1. Mathematical Modeling of Bionic Runner

Considering that the pipelines in animals and plants are highly similar to the pipe network system, and the internal fluid distribution and nutrient transport system performs tasks and functions with high efficiency, studying its basic structure can provide a design basis for optimizing pipelines.

**Figure 1:**
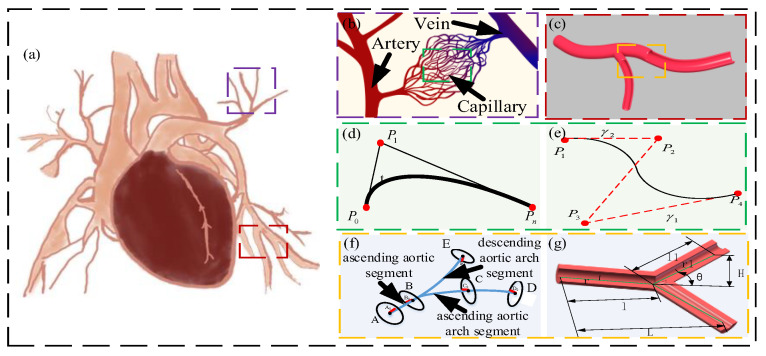
Flow path bionic mathematical modeling flow chart, (**a**) the human heart blood vessel diagram, (**b**) the arteriovenous connection network, (**c**) the fractal blood vessel model, (**d**) the Bessel curve of degree n, (**e**) the three-dimensional Bessel curve, (**f**) the fractal cardiovascular center line piecewise fitting diagram, (**g**) the internal structure of the vascular fractal model.

The cardiac vascular system is mainly composed of the heart, arteries, capillaries, and veins. As shown in Figure 1a, it is a closed circulation pipeline in which blood flows, supplying organs and tissues such as oxygen, various nutrients, and hormones. The arteries are connected between the heart and capillaries, transporting blood from the heart to the tissue. The capillaries are connected between the arteries and veins and connect each other into a network, as shown in Figure 1b.

The pipeline of the robot joint HDU is mainly composed of a servo valve inlet pipeline, a return pipeline, rod cavity pipeline, rod-less cavity pipeline, which can be used for reference of the arteriovenous layout of the cardiovascular system. In order to better extract the characteristics of vascular connection, the three-dimensional Bessel curve was used to fit it.

The Bessel curve changes the shape of the curve through the selection of control points. It is a trajectory curve composed of curve, endpoint, control point, and control line. Among them, the Bessel control line is a virtual line segment, which plays a key role in the trajectory generation process. According to the direction and length of the virtual line segment, the deflection direction and curvature radius of the Bessel curve can be changed, as shown in Figure 1d.

Usually, the *n* + 1 control point is defined to form a Bessel curve of the *n*th order, and its expression is:

(1) $$P(t)=\sum_{i=0}^{n}P_{i}B_{i,n}(t),t\in \left[0,1\right]$$

where $P_{i}$ represents the coordinate value of control point; t signifies parameters of control points; $B_{i,n}(t)$ denotes Bernstein polynomial.

Among them, the Bernstein polynomial $B_{i,n}(t)$ can be expressed as:

(2) $$B_{i,n}(t)=C_{n}^{i}t^{i}{\left(1-t\right)}^{n-i}$$

where $C_{n}^{i}$ represents the quadratic polynomial.

As shown in Figure 1f, the expression of the parametric equation of the three-dimensional Bessel curve composed of four control points can be expressed as:

(3) $$P(t)=P_{0}{\left(1-t\right)}^{3}+3P_{1}{\left(1-t\right)}^{2}t+3P_{2}(1-t)t^{2}+P_{3}t^{3}$$

where $P_{i}(x_{i},y_{i})$ denotes the horizontal and vertical coordinates of control points.

A vascular model in the cardiovascular system is selected, as shown in Figure 1c. This part of the vascular model assumes the role of transporting blood through fractals and a certain radius of curvature. Based on this feature, the required model is constructed.

Combined with the three-dimensional Bessel curve, the fractal blood vessel centerline is segmented and fitted from the three-dimensional level, and the fitting process is guaranteed to be simplified as much as possible under the premise of intuitive and flexible parameters. The constructed piecewise fitting centerline consists of four parts: ascending aortic segment, ascending aortic arch segment, descending aortic arch segment, and branch segment, as shown in Figure 1f.

The construction of the central line of the ascending aorta: the line segment from the starting point *A* to the adjacent point *A*_0_ is used as the line segment *AA*_0_ in the direction of the starting point of the ascending aorta. The ascending aorta and aortic arch junction point *B* was obtained according to the ascending aorta and aortic arch junction marker point *B*_0_. The only ascending aortic arc *AB* is constructed by the tangential vector at both ends of the arc and one of the ends.

Construction of the center line of the ascending aortic arch: From the junction point *B*_0_ between the ascending aorta and the aortic arch, according to the ascending vertex *C* of the aortic arch and the adjacent point *C*_0_, the line segment in the direction of the aortic arch top is constructed. Taking *B*_0_*C*_0_ as the endpoint, and *BB*_0_ and *CC*_0_ as the tangent direction, a three-dimensional Bessel curve is made. The fitting degree is strengthened by the distance between the two endpoints.

According to the construction method of (1) and (2), the construction of the center line of the descending segment of the aortic arch and the center line of the branch segment is completed. The proposed fitting curve provides a certain reference for the construction of bionic pipeline.

### 2.2. Channel Branch Angle Modeling

Murray’s law studies the relationship between different levels of vascular diameter, which assumes that the bifurcation network forms the most effective combination of the circulatory system through natural evolution. In the vascular bifurcation network system, the energy required to maintain blood flow consists of two parts: one is the energy required for blood flow $E_{f}$ , and the other is the energy required to maintain metabolism $E_{m}$ . To maintain the expression of blood energy flow:

(4) $$E_{f}=pq=\frac{128\mu lq^{2}}{\pi d^{4}}$$

where p represents the vascular pressure drop; q signifies the blood flow; μ denotes the blood viscosity coefficient; l represents the vascular length; and d depicts the vascular diameter.

The energy required to maintain metabolism $E_{m}$ increases with the increase in vascular length, which can be expressed as:

(5) $$E_{m}=mv=\frac{ml\pi d^{2}}{4}$$

where m represents the metabolic constant and v signifies the vascular volume.

The energy consumed by blood vessels can be obtained from Equations (5) and (6):

(6) $$E=E_{f}+E_{m}=\frac{128\mu lq^{2}}{\pi d^{4}}+\frac{ml\pi d^{2}}{4}$$

Assuming that the flow q and length l of the blood vessel are known, the energy consumption in the pipeline is related to the diameter d of the pipeline. Therefore, when the energy consumption is the smallest, it can be obtained:

(7) $$0=-\frac{512\mu lq^{2}}{\pi d^{5}}+\frac{ml\pi d}{2}$$

Then, the relationship between flow q and diameter d can be obtained by the following formula:

(8) $$q^{2}=\frac{m\pi^{2}d^{6}}{1024\mu }$$

For the branch pipeline on the aorta, a more reasonable assumption is that the flow q and the cross-sectional area of the branch pipeline s are constant, and the energy loss of the branch pipeline can be expressed as:

(9) $$E_{1}=\frac{128\mu s{q_{1}}^{2}}{\pi^{2}{d_{1}}^{5}}+\frac{msd_{1}}{4}$$

Similarly, when the energy consumption is minimal, the relationship between the flow q and the diameter $d_{1}$ is

(10) $${q_{1}}^{2}=\frac{m\pi^{2}{d_{1}}^{6}}{2560\mu }$$

Therefore, for arteries and branch vessels, there is a relationship:

(11) $$q=kd^{3}$$

where k is a constant. According to the law of conservation, there are the following relationships in a branch of a blood vessel:

(12) $$kd^{3}=k{d_{1}}^{3}+k{d_{2}}^{3}$$

Therefore, it can be deduced that in the vascular system, the size relationship between arterial vessels and branch vessels is

(13) $${r_{0}}^{3}={r_{1}}^{3}+{r_{2}}^{3}$$

where $r_{0}$ represents the arterial vascular radius; $r_{1}$ signifies the radius of branch vessel 1; and $r_{2}$ denotes the radius of branch vessel 2.

The blood vessel relationship in Formula (12) is also applicable to symmetric and asymmetric branches and also to the branch problem of circular pipelines, thus providing a direction for bionic pipeline design.

During the evolution of the visceral vascular system, its shape and size have reached the principle of energy optimization. Under this principle, according to the above analysis, a pipeline bionic vascular model is established, as shown in Figure 1g. Among them, q is the blood flow of the main blood vessel, and $q_{1}$ is the blood flow of the fractal pipeline. The radius relationship between the main blood vessel and the fractal blood vessel is studied, that is, the two main design parameters of $r/r_{1}$ and vascular angle θ .

As shown in Figure 1g, the main vessel length l and fractal vessels $l_{1}$ can be expressed as:

(14) $$\left\{\begin{array}{l} l=L-H/tg\theta \\ l_{1}=L-H/\sin\theta \end{array}\right.$$

According to Equations (6) and (14), the relationship between energy consumption and the radius of blood vessels can be obtained:

(15) $$E=\left(\frac{kq^{2}}{r^{4}}+k_{1}r^{\alpha }\right)\left(\frac{L-H}{\tan\theta }\right)+2\left(\frac{k{q_{1}}^{2}}{{r_{1}}^{4}}+k_{1}{r_{1}}^{\alpha }\right)\left(\frac{L-H}{\sin\theta }\right)$$

According to Equation (15), partial derivatives of $r,r_{1},\theta$ are obtained respectively:

(16) $$\left\{\begin{array}{l} \frac{\partial E}{\partial r}=0\\ \frac{\partial E}{\partial r_{1}}=0\\ \frac{\partial E}{\partial \theta }=0 \end{array}\right.$$

Formula (16) can further obtain the relationship between the radius and the angle between the blood vessels:

(17) $$\left\{\begin{array}{l} \frac{r}{r_{1}}=4^{\frac{1}{\alpha +4}}\\ \cos\theta =2{\left(\frac{r}{r_{1}}\right)}^{-4}=2^{\frac{\alpha -4}{\alpha +4}} \end{array}\right.,1\leq \alpha \leq 2$$

Finally, the design range of the radius and angle between blood vessels can be obtained:

(18) $$\left\{\begin{array}{l} 1.26\leq \frac{r}{r_{1}}\leq 1.32\\ 74^{\circ }\leq 2\theta \leq 98^{\circ } \end{array}\right.$$

Under the principle of bionic design, the calculation of the parameters of the typed blood vessel with the minimum energy is referred to. Through the above analysis and calculation, the calculation area of the radius and bifurcation angle of the blood vessel can be obtained when the number of fractals is 2, which can guide the bionic pipeline design.

### 2.3. Analysis of Pressure Loss in Channel Arrangement

**Figure 2:**
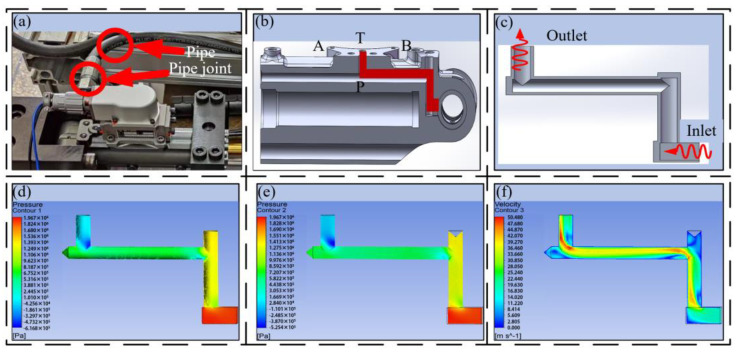
Traditional machining HDU flow channel layout and pressure velocity cloud diagram, (**a**) the traditional machining HDU, (**b**) the traditional machining HDU flow channel profile, (**c**) the traditional machining HDU flow channel layout, (**d**) the traditional machining HDU flow channel surface pressure nephogram, (**e**) the traditional machining HDU flow channel center pressure nephogram, (**f**) the traditional machining HDU flow channel center speed nephogram.

The traditional HDU pipeline connection usually adopts two methods. One is to set up a pipe joint on the shell to connect and transmit oil through an external hose, as shown in Figure 2a. With the continuous development of the HDU towards lightweight integration, the built-in pipeline is realized through machining. As shown in Figure 2b, this pipeline layout is limited by the processing method, resulting in a large number of the process holes, and the processed flow channels are mostly vertical connections. Its oil transfer performance needs further verification.

According to the pipeline section view shown in Figure 2b, because the oil enters the pipeline through the rotating oil distribution to reach the P port, it will pass through two vertical corners of 90°, and the pipeline connection is extracted to facilitate the understanding of the flow channel connection form.

According to the flow channel configuration shown in Figure 2c, the pressure loss is calculated and verified by theoretical calculation and simulation. Among them, the servo valve flow is 20 L/min.

The pressure loss can be expressed as

(19) hf=64Rel1d1v22g

where Re represents the Reynolds number; $l_{1}$ signifies the pipeline length; $d_{1}$ denotes the pipeline diameter; and v denotes the flow velocity.

The local pressure loss of 90° elbow is

(20) $$h_{\xi }=\xi \frac{\rho v^{2}}{2}$$

where ξ represents the local resistance coefficient of the 90° elbow.

The pressure loss of the reserved channel is

(21) $$h_{\xi_{1}}=\xi_{1}\frac{\rho v^{2}}{2}$$

where $\xi_{1}$ depicts the local resistance coefficient of the 90° elbow.

Therefore, the total pressure loss of the flow channel is

(22) $$\Delta h=\sum h_{f}+\sum h_{\xi }+\sum h_{\xi_{1}}$$

The total pressure loss of the flow channel is calculated to be 1.47 MPa. Combined with Figure 2c, the extracted pressure loss of the flow channel at the P port of the machining HDU is divided into the loss along the different lengths and the local loss from the right-angle bend. Due to the limitations of the processing form, different lengths of reserved channels will be formed at the corner of the flow channel during the processing of the flow channel. This part of the channel is easy to form eddy currents and other phenomena, resulting in greater local loss. In the calculation, this part is also simplified into the pressure loss caused by the elbow, and its accuracy is verified by subsequent simulation.

### 2.4. Establishment of Flow Channel Arrangement Model

The internal flow field of the traditional joint HDU is affected by the internal pipeline structure, resulting in a certain energy loss, which reduces the oil transfer efficiency from the servo valve to the inside of the shell. The pipeline arrangement extracted by the traditional HDU is mainly a Z-type channel structure. ANSYS Fluent is used to simulate the internal flow field of the pipeline, and the influence of the flow channel configuration on the oil velocity and pressure loss is studied. The ANSYS Fluent software, a fluid dynamics software under the ANSYS company, is utilized for simulating and analyzing fluid flow, heat transfer, and mass transport issues.

Figure 2d,e are the surface pressure nephograms of the P-channel flow channel of the machined HDU and the pressure nephograms at the central section of the flow channel. The hydraulic oil enters the pipeline at a certain initial speed and has a certain pressure. The greater the pressure, the faster the flow rate in the flow channel. At the oil outlet, there will be three sudden changes in direction. For each sudden change, the pressure of the oil will become smaller due to energy loss, and the flow rate will become slower. It can be observed from the center pressure nephogram of Figure 2e that the pressure attenuation will be more obvious. When the second direction is abruptly changed, the oil will also produce the phenomenon of wall attachment, resulting in a low pressure point below the pipeline, which is not conducive to the effective use of HDU energy. It can be seen from the simulation that the pressure loss of the oil through the traditional flow channel is 1.93 MPa. Due to the uncertainty of the local pressure loss, although there is a certain error with the calculation of Equation (22), it is enough to show that the pressure loss of the traditional flow channel is large.

Figure 2e is the flow channel center speed nephogram. It can be seen that the velocity of the oil at the inlet and outlet tends to be stable, and linear loss changes relatively smoothly. At the junction, due to the sudden change in direction, under the action of inertia, the oil will flow along the inner wall of the pipeline, and the oil velocity on the outer wall is relatively reduced. Due to the velocity difference, a slight secondary flow phenomenon is formed, resulting in increased wall friction loss. At the same time, due to the process hole formed by the processing technology, with the increase in its length, it will also lead to the obvious eddy current phenomenon of the oil at the corner and increase oil loss.

In general, the pressure loss of the pipeline with sharp turns is greater, so when we design the pipeline, the connection needs a smooth transition to reduce the loss and improve the energy transfer efficiency of the system. Therefore, it is necessary to optimize the large rotation angle of the joint HDU.

**Figure 3:**
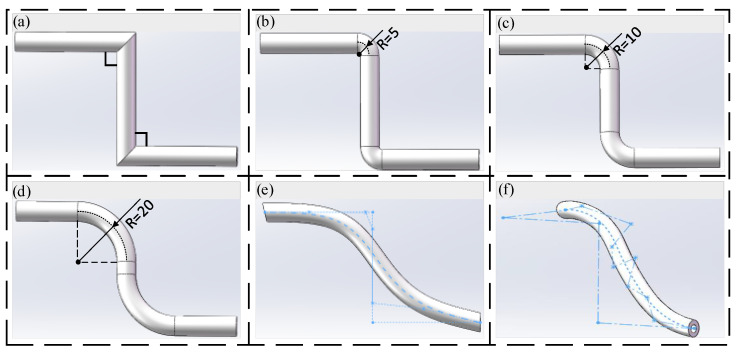
Models of the different pipeline transition modes, (**a**) linear pipeline model, (**b**) circular pipeline model with R = 5, (**c**) circular pipeline model with R = 10, (**d**) circular pipeline model with R = 20, (**e**) two-dimensional Bessel pipeline model, (**f**) three-dimensional Bessel pipeline model.

The flow channel designed by additive manufacturing technology is more flexible, and the pipeline arrangement also has more possibilities. Therefore, the performance optimization of the transition zone is based on the straight line, the arc transition curve, and the Bessel curve. Under the premise of ensuring that the inlet and outlet positions are unchanged, the models are constructed in SOLIDWORKS, as shown in Figure 3.

In order to better carry out simulation analysis, the simulation model adopts the same size as the joint HDU pipeline of the additive manufacturing version, that is, the inner diameter d = 3.8 mm and the wall thickness of 4 mm. In order to compare with the traditional HDU pipeline model, Figure 3a is a simplified model of Figure 2b. Figure 2a–e show pipeline models with the inlet and outlet on the same plane. Considering the characteristics of the additive manufacturing and that the HDU inlet and outlet are not on the same plane, the pipeline model formed by the three-dimensional Bessel curve is introduced.

## 3. Simulation Analysis of Flow Channel Pressure Loss

### 3.1. Flow Channel Optimization Model Simulation

The established three-dimensional model of the pipeline is imported into the ANSYS Fluent module for feature extraction and meshing, and the surface of the pipeline is meshed to form a 0.5 mm mesh. The simulation conditions are set as shown in Table 1.

**Figure 4:**
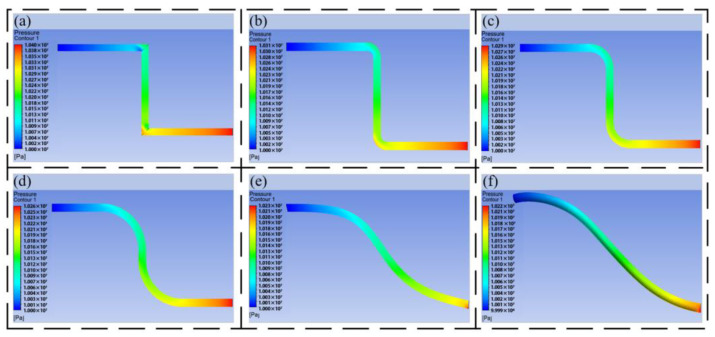
The pressure nephogram of the 10 m/s outlet of different three-dimensional models, (**a**) the pressure nephogram of the linear pipeline, (**b**) the pressure nephogram of the circular pipeline with R = 5, (**c**) the pressure nephogram of the circular pipeline with R = 10, (**d**) the pressure nephogram of the circular pipeline with R = 20, (**e**) the pressure nephogram of the two-dimensional Bessel pipeline, (**f**) the pressure nephogram of the three-dimensional Bessel pipeline.

The pressure nephogram and speed nephogram of the different flow channels are obtained by simulation, as shown in Figure 4.

Figure 4a–f are the pressure nephograms of the inlet velocity of 10 m/s and the outlet of 10 MPa under different transition modes. It can be seen from the figure that the oil has a large pressure loss at the right-angle corner, and the pressure change is obvious every time it passes through a right-angle corner. From Figure 4a–d, it can be seen that under the same outlet pressure, the inlet pressure is guaranteed to be about 10.2 MPa–10.3 MPa. As the transition radius gradually increases, the pressure loss gradually decreases. It can be seen that the arc transition can effectively avoid the pressure loss caused by the pipeline turning. It can be seen from Figure 4e,f that the Bessel curve introduced with reference to bionic experience can connect the outlet and inlet pipelines at different positions, which can make the flow channel smoother, which reflects the advantages of the Bessel curve in pipeline design.

**Figure 5:**
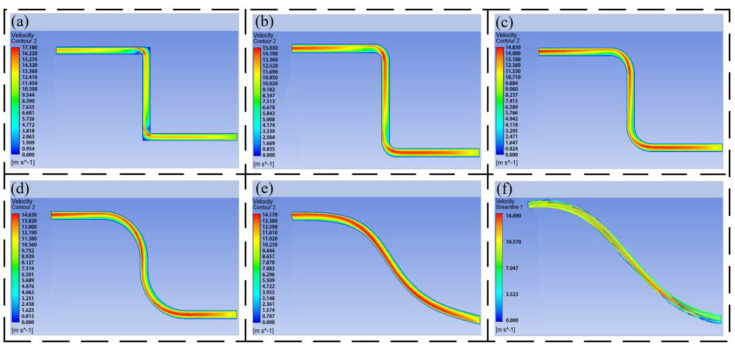
The speed nephogram of the 10 m/s outlet of different three-dimensional models, (**a**) the speed nephogram of the linear pipeline, (**b**) the speed nephogram of the circular pipeline with R = 5, (**c**) the speed nephogram of the circular pipeline with R = 10, (**d**) the speed nephogram of the circular pipeline with R = 20, (**e**) the speed nephogram of the two−dimensional Bessel pipeline, (**f**) the speed nephogram of the three-dimensional Bessel pipeline.

Figure 5a–f are the speed nephograms of the inlet at 10 m/s and the outlet at 10 MPa under different transition modes. It can be seen from the figure that the velocity of the oil passing through the right-angle turn will change dramatically. The velocity inside the right angle is low, and the velocity outside the right angle is large. With the increase in the inner diameter of the pipeline, it is easy to form an eddy current, resulting in energy loss. Compared with Figure 5b–f, the flow characteristics of the oil in the pipeline have been greatly improved compared with Figure 5a, and with the increase in the arc, the flow performance is better, and half of the oil at the outlet of the pipeline can reach the maximum speed. The flow characteristics of the oil in the pipeline formed by the Bessel curve shown in Figure 5e,f are the best, which indicates that the HDU pipeline based on additive manufacturing can be designed by using the large arc and Bessel spline curve.

### 3.2. Analysis of Pressure Loss in Flow Channel

**Figure 6:**
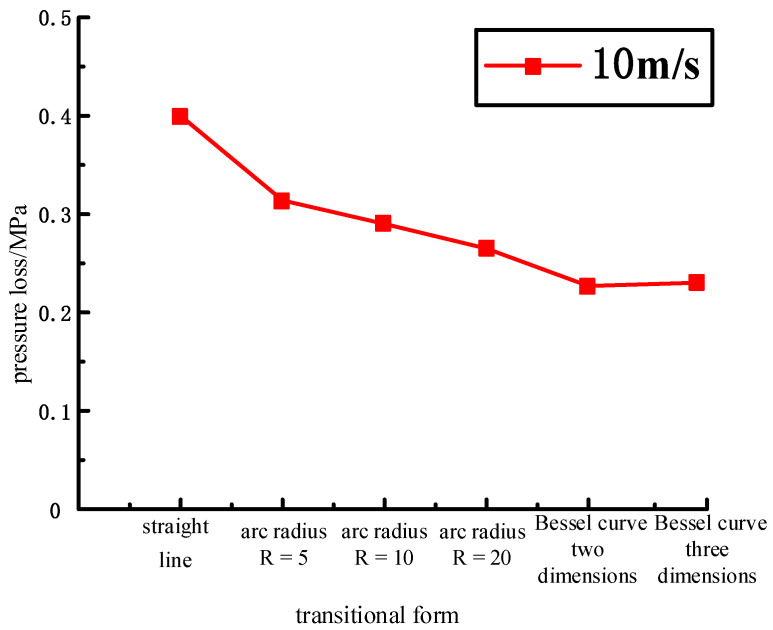
Comparison of pressure loss under different transition modes at the outlet 10 m/s.

At the same time, quantitative analysis was conducted on the pressure loss under different transition modes, as shown in Figure 6 and Table 2.

Under the same boundary conditions, the pressure loss of linear transition is the largest, the pressure loss of arc transition and Bessel transition is small, and the pressure loss decreases with the increase in arc radius. At the same time, the inlet and outlet of the pipeline are not on the same plane, and the pressure loss generated by Bessel is close. From the data in the table, it can be seen that the pressure loss of the arc radius R = 5 pipeline is 21.5% lower than that of the linear pipeline, the pressure loss of the arc radius R = 10 pipeline is 27.5% lower than that of the linear pipeline, the pressure loss of the arc radius R = 20 pipeline is 33.75% lower than that of the linear pipeline, and the pressure loss of the pipeline with different forms of Bessel curve is 40–45% lower than that of the linear pipeline. In summary, for the design of the HDU pipeline transition area, the use of large arc transitions and Bessel transitions significantly reduces the pressure loss of the flow channel, so that the oil in the pipeline has good flow characteristics.

## 4. Additive Manufacturing Version Joint HDU Flow Channel Layout

### 4.1. Channel Arrangement of HDU

In order to better determine the overall scheme of the joint HDU pipeline of the additive manufacturing version, the overall analysis of the HDU pipeline is carried out, and then the bionic pipeline layout is applied to the shell.

**Figure 7:**
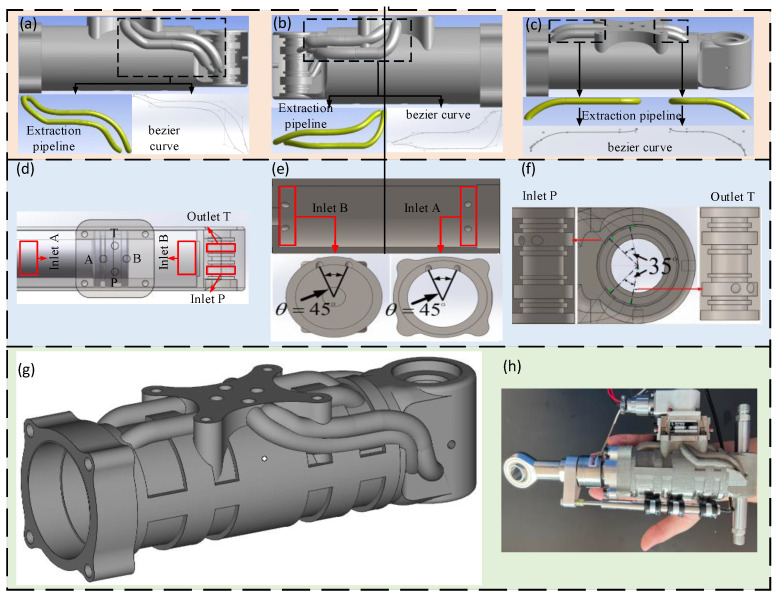
Additive manufacturing joint HDU pipeline shape, position, and HDU three-dimensional structure diagram, (**a**) the pipeline at P, (**b**) the pipeline at T, (**c**) the pipeline at AB, (**d**) the HDU pipeline port position, (**e**) the joint HDU shell pipeline inlet design, (**f**) the joint HDU tail end rotating oil distribution structure pipeline, (**g**) joint HDU three-dimensional structure, (**h**) additive manufacturing joint HDU.

The pipeline at the P oil port of the joint HDU of the additive manufacturing version is mainly connected with the rotary oil distribution to realize the external oil source to provide oil to the actuator. Its pipeline layout is shown in Figure 7a,b. Because the inlet and outlet of the oil circuit are not on the same plane, the annular cavity of the rotating oil distribution is fractal, and the corresponding oil port of the servo valve installation platform is connected by the Bessel curve–straight line–Bessel curve.

The A oil port of the joint HDU is mainly connected with the rod cavity, and port B is connected to the rod-less cavity, which is used to realize the horizontal movement of the piston and the output of the actuator. Its pipeline layout is shown in Figure 7c. The shell is regarded as a ring-shaped cavity, which is connected to the corresponding oil port of the servo valve installation platform by the Bessel curve–straight line–arc line.

### 4.2. Comparative Analysis of Flow Channel Performance

**Figure 8:**
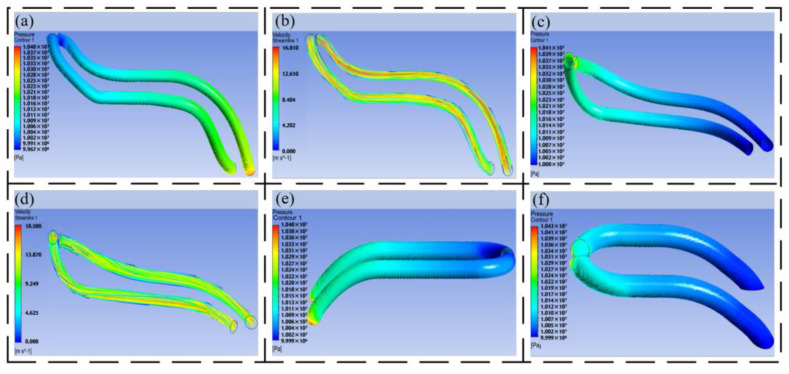
Simulation analysis of each pipeline of the HDU of the additive manufacturing version, (**a**) the pipeline pressure nephogram at P, (**b**) the pipeline speed nephogram at P, (**c**) the pipeline pressure nephogram at T, (**d**) the pipeline speed nephogram at T, (**e**) the pipeline pressure nephogram at A, and (**f**) the pipeline pressure nephogram at B.

After completing the design of the bionic pipeline of the additive manufacturing joint actuator, the oil performance in the pipeline is analyzed by ANSYS Fluent, as shown in Figure 8.

**Figure 9:**
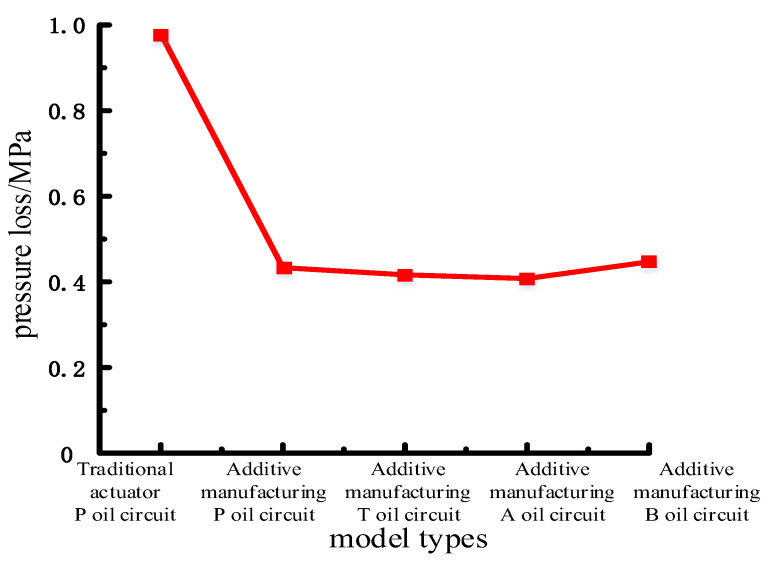
Pressure loss of each pipeline of the joint HDU of the additive manufacturing version.

The pressure loss of each pipeline is quantitatively analyzed, as shown in Table 3 and Figure 9, and compared with the traditional HDU pipeline pressure nephogram shown in Figure 2a.

From the pressure nephogram shown in Figure 8, it can be seen that the pressure loss of the traditional HDU pipeline is 0.98 MPa, and the overall pressure loss of the pipeline after the optimization of each pipeline of the joint HDU of the additive manufacturing version is 0.40 MPa–0.45 MPa. After optimization, the pressure loss of oil in the pipeline is greatly reduced. From the data in Table 3, it can be seen that the pipeline pressure loss at P of the additive manufacturing version is 55.9% lower than that of the traditional pipeline, the pipeline pressure loss at T of the additive manufacturing version is 57.6% lower than that of the traditional pipeline, the pipeline pressure loss at A of the additive manufacturing version is 58.5% lower than that of the traditional pipeline, and the pipeline pressure loss at B of the additive manufacturing version is 54.5% lower than that of the traditional pipeline. The optimized pipeline can improve the energy transmission efficiency of the HDU by more than 50%.

According to the branch angle parameters of the bionic flow channel, the main oil circuit connection block of the joint HDU is integrated to obtain the rotating oil distribution structure, which is distributed with the oil inlet P and the oil return port T, and the HDU shell is distributed with the rod cavity oil port A and the rod less cavity oil port B. The above four oil port positions are shown in Figure 8d.

The oil inlet P and the oil return port T at the tail end of the rotating oil distribution are designed. According to the range of the bifurcation angle of the bionic pipeline, the two oil ports are designed according to the $\theta =35^{\circ }$ . Under the ring cavity structure of the rotating oil distribution, the oil supply efficiency of the servo valve to the HDU shell is improved, and the structure of the rotating oil distribution pipeline at the end of the joint HDU is obtained.

As shown in Figure 8e, the rod cavity oil port A and the rod cavity oil port B on the shell are designed. Similar to the ring cavity of rotating oil distribution, the HDU shell itself is a ring cavity with a larger inner diameter, and there is more oil passing through the A and B ports of the pipeline per unit time. Therefore, a larger angle of pipeline fractal is selected, that is $\theta =45^{\circ }$ , to ensure that the oil can have a smaller loss after entering the pipeline.

The final three-dimensional structure of the joint HDU is obtained by combining the bionic flow channel and the main oil circuit rotating oil distribution structure, as shown in Figure 7h.

## 5. Experimental Verification

To better test the superiority of the bionic channel in the HDU and the lightweight effect of the prototype, the corresponding bionic channel is extracted to verify its performance.

Two joint HDU prototypes are manufactured by machining and additive manufacturing technology, respectively, as shown in Figure 2a and Figure 7g. The flow channel of the machined HDU is arranged in a traditional way, while the additive manufacturing joint HDU adopts the form of the bionic flow channel to prepare for the subsequent flow channel extraction and performance test.

**Figure 10:**
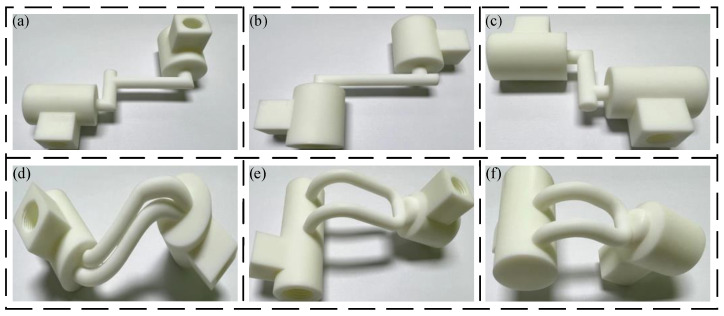
HDU flow channel of machining version and additive manufacturing version, (**a**) P flow channel of machining version named Pipe 1, (**b**) A flow channel of machining version named Pipe 2, (**c**) B flow channel of machining version named Pipe 3, (**d**) P flow channel of additive manufacturing version named Pipe 4, (**e**) A flow channel of additive manufacturing version named Pipe 5, (**f**) B flow channel of additive manufacturing version named Pipe 6.

The four pipelines in the two joint actuators were separately extracted, modeled, and manufactured. The inner diameter, wall thickness, length, and rotation angle of the flow channel were guaranteed to be identical during the processing. Because there is no difference in the structural parameters between the shell of the two HDU prototypes and the oil inlet and return channels of the servo valve in the design process, only the oil inlet channel of the servo valve (P channel), the rod cavity flow channel of the shell (A channel), and the rod less cavity flow channel (B channel) are verified. The flow channels processed and produced are shown in Figure 10.

As shown in Figure 10, the cylindrical cavities with the positive inlet and outlet of the flow channel are respectively arranged at both ends of the flow channel, which are used to connect with the inlet and outlet hoses of the hydraulic system during the test. At the same time, there is a square structure at the upper end of the cylindrical cavity. The upper end is connected to the cylindrical cavity through a threaded hole, which is used to install a pressure sensor to detect the pressure at both ends of different flow channels.

**Figure 11:**
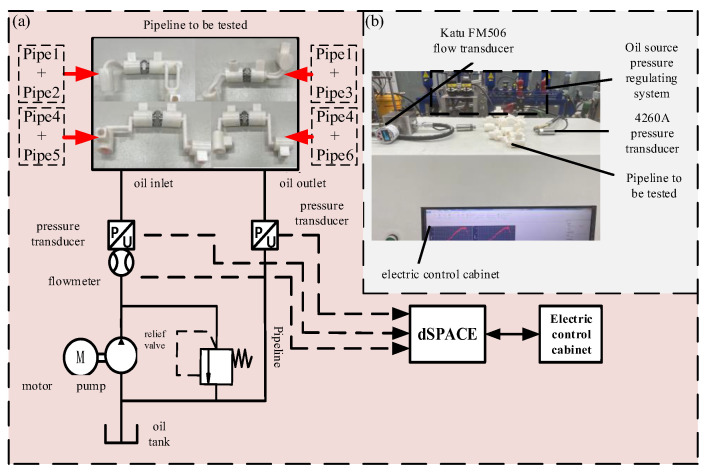
Pressure loss test platform principle and physical diagram, (**a**) the pressure loss test platform schematic diagram, (**b**) the pressure loss test platform diagram.

In order to complete the pressure loss test of the prototype flow channel more accurately, a pressure loss test platform was built. The principle is shown in Figure 11a, which mainly includes three parts: the oil source pressure regulation system, the pipeline to be tested, and the control acquisition system.

The pressure loss test platform is shown in Figure 11b. The control acquisition system is based on dSPACE. The data are collected and stored by the program block diagram written by MATLAB.

In order to better verify the flow channel optimization results, the P, A, and B flow channels of the two joint actuators were tested separately because the T and P flow channels of the HDU are the same in the actual process, so they are not tested separately. At the same time, in order to better meet the real working state of the HDU flow channel, the P-A flow channel is combined and tested to simulate the extended state of the HDU piston rod; the P-B flow channel combination test simulates the state of the HDU piston rod retraction.

During the test, the flow range is 3–15 L/min, the relief valve is adjusted to realize the flow change in different sizes in the pipeline, and the inlet and outlet pressure of the flow channel is read by the pressure sensor at both ends of the flow channel. Then, the pressure difference is used to determine the pressure loss of different flow channels.

The average value of the collected data is taken, and the flow-pressure difference curve is drawn. The error of the experimental results is defined as:

(23) $$\sigma =\sqrt{\frac{\sum_{i=1}^{n}{\left(x_{i}-\bar{x}\right)}^{2}}{N}}$$

**Figure 12:**
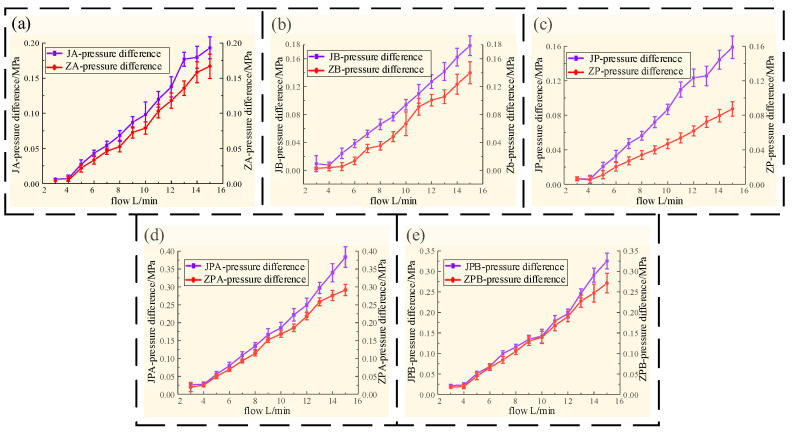
Different forms of flow channel pressure loss, (**a**) the flow channel pressure loss at A in two ways, (**b**) the flow channel pressure loss at B in two ways, (**c**) the flow channel pressure loss at P in two ways, (**d**) the flow channel pressure loss at P-A in two ways, (**e**) the flow channel pressure loss at B-P in two ways.

The pressure loss test results of different flow channels are shown in Figure 12.

It can be seen from Figure 12 that during the experiment, the pressure difference on both sides of the flow channel increases with the increase in the flow rate, and the pressure difference on both sides of the flow channel of each section of the machined joint HDU is higher than that of the additive manufacturing joint actuator.

It can be seen from Figure 12a,b that through the pressure loss experiment of the AB pipeline, the loss along the path caused by the shape of the flow channel is not obvious at a small flow rate. When the flow rate reaches 15 L/min, the maximum pressure loss of machining is 0.015 MPa higher than that of additive manufacturing. It can be seen that although the length of the AB flow channel is short, the pressure loss is obviously reduced by optimizing the angle of the AB pipeline.

From the pressure loss curve of the P channel in Figure 12c, it can be found that as the flow rate gradually increases, the growth rate of the P channel pressure difference in additive manufacturing is slower than that of the machining channel. When the flow rate reaches 15 L/min, the maximum pressure loss of machining is 0.035 MPa higher than that of additive manufacturing. From Figure 12d,e, it is found that the pressure loss of the additive manufacturing flow channel is generally lower than that of the machining flow channel during the extension and retraction of the HDU piston rod, and the larger the flow rate, the more obvious the effect of reducing the pressure loss. When the flow rate reaches 15 L/min, the maximum pressure loss of machining is 0.09 MPa higher than that of additive manufacturing.

Through the pressure loss at each stage in the above diagram, the average pressure loss and average error of each flow channel are obtained. The detailed data are shown in Table 4.

According to the analysis in Table 4, the bionic flow channel formed by additive manufacturing is obviously better than the machined version in reducing the loss along the path, and the data error is smaller. The overall optimization ratio is also more obvious with the increase in pipeline length, and the optimization ratio of the P flow channel reaches 45%. Although the pressure loss of the combined flow channel is slightly improved due to the limitations of the actual structure, the optimized flow channel can directly improve the oil transfer efficiency, which proves that the bionic flow channel has an obvious effect on reducing the pressure loss and improving the oil transfer efficiency.

## 6. Conclusions

Based on the additive manufacturing technology, this paper uses the Bessel curve to model human heart blood vessels and determines the branch angle and radius ratio of the bionic flow channel based on the Murray criterion.

Through ANSYS simulation analysis, it is verified that the pressure loss of the designed bionic flow channel is reduced by more than 40% compared with the machining form, which verifies the feasibility and advancement of the flow channel optimization of the joint HDU in the additive manufacturing version.

Finally, a pressure loss test platform was built, and the pressure loss curves of the machining flow channel and the bionic flow channel were obtained. It can be seen from the calculation that the pressure loss of the bionic flow channel is reduced by 45% compared with the machining form, which proves that the bionic flow channel has an obvious effect on reducing the pressure loss. Based on the design concept of the bionic flow channel, an additive manufacturing joint HDU prototype was formed. It can be applied to various joints of the legs of legged robots to improve their motion performance. In the future, the HDU prototypes developed in this study can be separately employed across the joints of quadrupedal robots, thereby enhancing robot efficiency by reducing pressure loss. Additionally, we can contemplate further reducing the weight of the HDU by drawing inspiration from structures found in other biological organisms.

## Figures and Tables

**Table 1:** Simulation initial conditions.

Simulation Parameter	Boundary Name	Boundary Types	Related Parameters
	inlet	Velocity, Pressure	10 m/s, 10 MPa
		Velocity, Pressure	10 m/s, 10 MPa
	outlet 1	——	Q_1_, P_1_
	outlet 2	——	Q_2_, P_2_
46# resistance of abrasion hydraulic fluid	density	——	860 kg/m^3^
	kinematic viscosity	——	0.03956 m^2^/s

**Table 2:** Comparison of pressure loss under different transition modes at the outlet 10 m/s.

Boundary Condition	Model Types	Inlet Pressure/MPa	Outlet Pressure/MPa	Pressure Loss/MPa	Compared with the Linear Transition Pressure Loss Reduction Percentage/%
10 m/s	straight line	10.4	10	0.4	
	R = 5	10.314	10	0.314	21.5
	R = 10	10.29	10	0.29	27.5
	R = 20	10.265	10	0.265	33.75
	YT1	10.227	10	0.227	43.25
	YT2	10.22	9.99	0.23	42.5

**Table 3:** Comparison of pressure loss of the additive manufacturing actuator.

Boundary Condition	Model Types	Inlet Pressure/MPa	Outlet Pressure/MPa	Pressure Loss/MPa	Compared with the Linear Transition Pressure Loss Reduction Percentage/%
10 m/s	Traditional pipeline	10.7852	9.8034	0.9818	
	Pipeline P	10.4003	9.967	0.4333	55.9
	Pipeline T	10.4163	9.9998	0.4165	57.6
	Pipeline A	10.404	9.9962	0.4078	58.5
	Pipeline B	10.4369	9.99	0.4469	54.5

**Table 4:** Pressure loss data analysis of different flow channels.

Test Flow Channel	Pressure Difference/MPa		Data Error σ		Additive Manufacturing
	Machining	Additive Manufacturing	Machining	Additive Manufacturing	
Flow channel A	0.0951	0.0883	0.0095	0.0085	16.3%
Flow channel B	0.0836	0.0590	0.0091	0.0088	29.4%
Flow channel P	0.0762	0.0419	0.0074	0.0053	45.0%
Flow channel PA	0.1751	0.1478	0.0146	0.0093	15.6%
Flow channel PB	0.1457	0.1315	0.0102	0.0117	9.7%

## Data Availability

The data presented in this study are available in the main text.
